# The robustness of repetition-based illusory truth and certainty effects in social media contexts

**DOI:** 10.1038/s41598-026-61449-y

**Published:** 2026-08-05

**Authors:** Annika Stump, Raphael Hartmann, Karl Christoph Klauer

**Affiliations:** Institute of Psychology, University of Freiburg, Engelbergerstraße 41, 79085 Freiburg im Breisgau, Germany

**Keywords:** Truth judgments, Subjective confidence, Information repetition, Social media, Social endorsement cues, Likes, Neuroscience, Psychology, Psychology

## Abstract

In the past, cognitive illusions, such as the repetition-based illusory truth and certainty effects, were primarily studied in experimental settings that were far removed from real-world conditions. However, information channels – particularly social media – provide competing cues (e.g., likes) that may influence such cognitive phenomena. In the present study (*N* = 165), participants were assigned to one of two groups that viewed Instagram-like posts either with visible like counts (experimental condition) or without them (control condition). In addition, within the experimental group (*N* = 82), the number of likes was systematically manipulated. Results showed no moderating effect of like visibility on the repetition-based illusory truth or certainty effects. However, especially when repetition-based cues were absent, low (compared to high) like counts substantially reduced both subjective truth and confidence (experimental condition), highlighting the dominant role of information repetition in shaping perceived truth and confidence in truth evaluations.

## Introduction

In modern digital environments, we are continuously exposed to an overwhelming stream of information that shapes our opinions, attitudes, and behavior. However, not all information encountered is valid, and under conditions of uncertainty, people frequently rely on mental shortcuts to evaluate credibility^1^. Such heuristics often draw on subjective experiences that accompany information processing, such as perceived ease or affective tone. While those heuristics can indeed serve as useful cues when validity is difficult to judge^2^, relying on these internal cues can also lead to systematic biases, or cognitive illusions.

One of the most robust examples of such a bias is the illusory truth effect – the tendency for repeated information to be judged as more truthful than novel ones^3,4^. This effect is largely attributed to processing fluency, the subjective experience of ease when processing repeated (vs. new) information. When information is processed fluently, individuals are more likely to perceive it as correct – a phenomenon that has been shown to arise from manipulations of both perceptual (e.g., Reber and Schwarz,^5^) and conceptual fluency (e.g., Arkes et al.^6^). In line with this, more recently, diffusion-model analyses suggest that the repetition-based truth effect may reflect a combination of perceptual facilitation, a subsequent bias toward affirming repeated statements, and increased speed of information accumulation supporting the truthfulness of the presented information^7^. Research further indicates that this subjective experience of ease is usually accompanied by a positive affective component (see e.g., Topolinski et al.^8^, Winkielman^9,10^), which is also the case in the context of the repetition-based illusory truth effect^11,12^. Beyond fluency, several studies have shown that positive affective states themselves can evoke a sense of familiarity and induce subjective truth. For instance, Garcia-Marques et al.^13^ found that perceived positivity alone can signal familiarity (Experiment 2) and that a positive (vs. neutral) affective state increases the likelihood of judging statements as true (Experiment 3). Similarly, and consistent with the idea that individuals match their current affective states with affectively consistent judgments (see, e.g., the affective matching model,^14,15^, current research suggests that judging information as true (vs. false) is systematically related to increased positive affect^16^.

Recent work has further shown that repetition affects not only judgments of truth but also the subjective confidence with which these judgments are made. This phenomenon is referred to as the illusory certainty effect^17^. The illusory certainty effect describes an increase in individuals’ subjective confidence in their judgments as a function of repetition, independent of whether those judgments are objectively correct. Thus, repeated information is not only more likely to be judged as true, but these judgments are also held with greater subjective certainty. Compared to research on truth judgments, subjective confidence in these judgments has received relatively little attention. The illusory certainty effect represents an initial example of how cognitive shortcuts – such as repetition – not only shape immediate truth judgments but also the confidence with which these judgments are held. It is plausible that this is not an isolated phenomenon restricted to repetition-based effects but rather reflects a more general mechanism that deserves increased attention in future research. At the same time, the cognitive mechanisms underlying the illusory certainty effect remain comparatively underexplored, including whether repetition-based fluency plays a similarly central role in the illusory certainty effect as has been proposed for the illusory truth effect.

To date, most studies investigating these repetition-based illusions have been conducted in highly controlled laboratory settings. However, real-world information environments – particularly social media platforms – differ in crucial ways. Online, information is often accompanied by social endorsement cues such as the number of likes or shares, which themselves serve as heuristics for truth^18^, and possibly for confidence judgments as well. Thus, social endorsement cues – such as likes – may shape repetition-based illusions of truth and certainty.

The present study seeks to examine how repetition-induced truth and certainty effects occur under more naturalistic, social media-like conditions. Specifically, we investigate whether the number of likes on social media posts influences (a) truth and confidence judgments as well as (b) repetition-based illusory truth and certainty effects. To this end, participants viewed Instagram-style posts that vary in both repetition status (repeated vs. new) and like count (high vs. low). We furthermore implemented two experimental groups: While one group of participants was exposed to posts that included visible likes, another group viewed the same posts without such information. This design allowed us to examine not only the effect of the number of likes but also the impact of the mere presence of likes as a competing heuristic cue. First, the mere presence of social endorsement cues (i.e., information is presented with vs. without likes) may already diminish reliance on repetition by providing a more salient cue for subjective truth and certainty. In this case, repetition may no longer be used as a cue for truth and certainty judgments, thereby preventing repetition-based effects from occurring. Second, the magnitude of social endorsement cues (i.e., high vs. low numbers of likes) may directly influence truth and certainty judgments as well as potentially moderate the repetition-based illusory truth and certainty effects.

We expected repeated statements to have a higher likelihood to be judged as true (vs. false) and with greater confidence than novel ones, replicating the classic illusory truth and certainty effects. Posts accompanied by high numbers of likes are expected to have a higher likelihood to be judged as true (vs. false) and with a higher confidence than those with few likes, reflecting the influence of social endorsement as a heuristic cue. Furthermore, because likes may provide an alternative source for judgements of truth and validation, their mere presence could reduce or eliminate the effects of repetition.

It is worth noting that the influence of social endorsement cues (such as likes) on truth judgments represents a relatively recent line of research, and the underlying processes are still insufficiently understood. Different mechanisms may be involved. For instance, high numbers of likes may elicit positive affect but may also function more generally as epistemic signals, for example in terms of social consensus or shared knowledge. Accordingly, it remains unclear how such social endorsement cues relate to the mechanisms associated with repetition-based effects, such as processing fluency, familiarity, or affect. We therefore do not assume that likes influence all components of the repetition-based effect equally, nor do we derive specific process-level hypotheses. (Previous research suggests that contextual factors such as expecting to encounter falsehoods can reduce reliance on repetition and thereby diminish illusory truth effects^19^. However, in the context of social endorsement cues, it remains unclear whether high numbers of likes serve as epistemic signals of social consensus or shared knowledge – and therefore may influence reliance on repetition – or whether they instead primarily function through other mechanisms, such as eliciting affective reactions. Instead, the present research investigates whether social endorsement cues influence repetition-based illusory truth and certainty effects in the first place.

Taken together, by embedding well-established cognitive mechanisms within ecologically more valid social media environments, this study aims to deepen our understanding of how truth and confidence judgments are shaped by repetition and social endorsement cues. This will strengthen our understanding of how (mis-)information can be spread and how people’s beliefs can be modulated in digital societies.

## Method

We analyzed data from a larger study which was designed to address several research questions. Informed consent was obtained from all study participants. The study was conducted in accordance with the Declaration of Helsinki. It employed standard procedures investigating the illusory truth effect that had previously received approval from the Ethics Committee of Heidelberg University (Faculty of Behavioural and Cultural Studies). As the present study involved only minor modifications of this established paradigm (i.e., the addition of like-count information) and did not introduce any new ethical considerations, no separate ethics approval was obtained. In the following, we describe all procedures and measures that are relevant to the present hypotheses. The study was not preregistered.

### Participants and design

A total of 165 individuals participated in the study, recruited via Prolific. In multilevel analyses, the level-2 sample size is typically most critical for statistical power^20^. Simulation studies by Maas and Hox^20^ indicate that parameter estimates become sufficiently accurate when at least 50 level-2 units are included (see also^21^). Similar recommendations have been made for multilevel logistic regression models, suggesting that a minimum of 50 level-2 units is required to obtain valid estimates^22^. In the present study, level-2 units refer to participants. Following these guidelines and to ensure comparability with previous truth effect studies (e.g., Stump et al.^17^; including 75 level-2 units), we aimed to recruit 75 participants per group (i.e., control vs. experimental group). To account for potential exclusions, we planned to oversample by approximately 10%, resulting in a total target sample size of *N* = 165. As no participants had to be excluded, the final sample consisted of *N* = 83 in the control group (participants exposed to posts without likes) and *N* = 82 in the experimental group (participants exposed to posts with likes). Participants ranged in age from 19 to 72 years (*M* = 31.90, *SD* = 10.17). 31% of the sample identified as female, 67% as male, and 2% as diverse. Compensation amounted to £3 (approximately £9 per hour).

Two experimental groups were realized: one serving as a control group and one exposed to posts with visible like counts. In the experimental condition, the design included two within-subject factors – r*epetition status* (new vs. repeated) and *likes* (low vs. high) – whereas in the control group, only repetition status was manipulated within participants.

### Material

The main stimulus set comprised 80 statements in German language, divided into two sets. To reduce potential primacy and recency effects, 12 additional statements (the first and last six statements during the exposure phase) were included. Another eight statements served as practice trials prior to the experimental judgment phase. Previous research has shown that although repetition-induced processing fluency itself may arise automatically, the interpretation as well as use of this experience for truth judgments is not^23^. In line with that finding, more recent work suggests that people rely on repetition particularly when relevant knowledge is unavailable or difficult to retrieve (e.g., Shechter and Klauer^24^). Accordingly, we used statements by Nadarevic^25^ that had been pretested to be sufficiently difficult (i.e., showing moderate mean truth ratings around the center of a Likert scale ranging from “definitely false” to “definitely true” and similar standard deviations, indicating relatively high uncertainty regarding their validity) and to reliably elicit an illusory truth effect in prior studies. Only affectively neutral statements were included, as their content should not trigger any affective reactions. The statements varied across several content domains, including geography, biology, politics and history, science, and entertainment. Examples of such statements are “A beaver can fell more than 200 trees in a year” (translated from the original German material; true) and “Women get hiccups more often than men” (translated from the original German material; false). All statements were matched in length to ensure comparable reading times. The statements had a mean length of *M* = 7.34 words (*SD* = 1.71). In terms of character count, the statements had a mean length of *M* = 49.60 characters (*SD* = 8.28). True statements had a mean length of 7.25 words (*SD* = 1.60) and 48.48 characters (*SD* = 8.10), whereas false statements had a mean length of 7.43 words (*SD* = 1.84) and 50.73 characters (*SD* = 8.40). All statements used in the present study can be found in the dataset provided on the OSF repository. The statements were organized into two sets containing 20 true and 20 false statements each. During the judgment phase, statements from both sets were presented: one consisting of items previously shown during the exposure phase and one comprising new items. The assignment of statement sets to experimental phases was counterbalanced across participants. In the experimental group, posts were displayed with visible like counts. 50% of the true and false statements within each statement set were presented with high (vs. low) numbers of likes. High-like posts displayed randomly generated counts between 20,000 and 80,000, while low-like posts ranged between 500 and 2,000. All posts were designed to match the visual layout of the current Instagram interface.

### Procedure

After providing informed consent, participants completed the online experiment programmed in lab.js^26^. In the first phase, 52 statements were presented one by one (without like counts), and participants rated each statement for interest on a 6-point scale (from “1 = not at all interesting” to “6 = very interesting”). The initial and final six trials served as buffers against primacy and recency effects and were identical for all participants. The 40 statements in between (20 true/20 false) were presented in random order. Each trial began with a 1000 ms fixation cross, followed by a statement displayed until the participant responded. Although no time limit was imposed, participants were asked to respond quickly but accurately.

In a subsequent 5-minute retention interval, the subjects worked on a non-verbal filler task consisting of alternating blocks of a go/no-go task (i.e., responding as quickly as possible to green and red circles using designated keys) and symmetry judgments of geometric shapes. Thereafter, the judgment phase of the experiment started. During the judgment phase, 40 novel and 40 repeated statements were shown trial-by-trial in random order in the form of Instagram posts. The 40 new statements were taken from the previously unused statement set, while the 40 repeated statements originated from the set presented during the exposure phase. Each trial started again with a fixation cross that was displayed for 1000 ms. The subsequently presented statement remained on the screen until participants gave their truth judgment by pressing the “W” key (“wahr”, German for true) or “F” key (“falsch”, German for false). Afterwards, participants rated the confidence with which their previous truth judgment was made by pressing one of the upper number keys (1 “very uncertain” to 6 “very certain”). There was no time limit for judgments. However, as in the exposition phase, subjects were instructed to make their judgments as quickly as possible, without avoidable mistakes.

At the end of the experiment, participants were additionally asked to respond to three brief questions concerning their social media use. Specifically, they indicated (a) whether they use social media platforms such as Instagram or Facebook (agree/disagree), (b) whether they currently use or have previously used Instagram (agree/disagree), and (c) how frequently they use social media platforms (e.g., Instagram, Facebook; 1 = not at all to 6 = several times a day).

## Results

To test our hypotheses, we estimated a series of multilevel models examining the effects of repetition status, like visibility, and like count magnitude on (i) truth judgments and (ii) confidence ratings. Accordingly, the results section is organized into analyses of truth judgments and confidence ratings. Within these sections, we first tested whether repeated statements were more likely to be judged as true and with greater confidence than new statements, thereby replicating the classic illusory truth and certainty effects. Second, we investigated whether the presence of likes reduced the repetition-based effects. Third, we examined whether social endorsement cues in the form of high (vs. low) like counts increased truth judgments and confidence ratings and whether they moderated the repetition-based effects. Following a reviewer suggestion, we additionally estimated exploratory mixed-effects models across the full sample including all three levels of social endorsement information (no likes, low likes, high likes). These analyses yielded a pattern of results consistent with the primary analyses reported below and are provided in Appendix A.

A multilevel modeling approach was used for all regression analyses to account for the hierarchical structure of the data. The analyses were performed with the statistical software R (version 4.4.2) using the lme4 package^27^ in combination with the lmerTest package^28^ for calculating p-values. All models included random intercepts for subjects. In models including the level-1 predictors repetition and like number, random slopes were also specified for these predictors. The predictors repetition status and like number were dummy coded, with new (vs. repeated) statements and the high (vs. low) likes serving as the reference categories, respectively.

Because heuristic cues (i.e., repetition and likes) were assumed to be used particularly under conditions of uncertainty, the statements were intentionally selected to be highly difficult (see Material section), thereby minimizing the contribution of factual knowledge to participants’ judgments. Accordingly, factual truth status was not included in the primary regression analyses. Exploratory analyses based on models including factual truth status and its interactions repeatedly showed convergence problems, supporting the use of the more parsimonious models reported below. A further exploratory analysis included factual truth status as a sole predictor for truth judgments. The results revealed no significant effect of factual truth (*b* = 0.043, *p* = .268), indicating that knowledge did not play a substantial role in the present study.

To further investigate the potential cognitive processes underlying the effects of likes and repetition on judgments of truth, we conducted an exploratory model comparison of response-time extended multinomial processing tree models^29^.

### Truth judgments

Truth judgments were analyzed using a generalized linear mixed model based on maximum likelihood (Laplace approximation). To account for the dichotomy of the judgments (true vs. false), a logit link function was used.

### The illusory truth effect as a function of like visibility

**Fig. 1:**
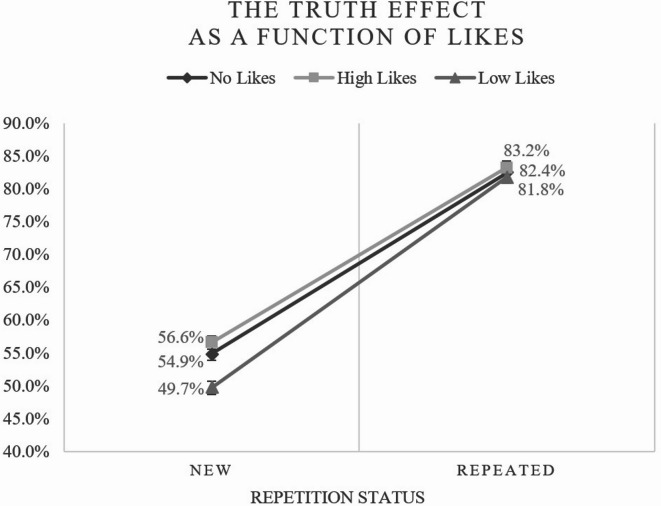
The illusory truth effect as a function of likes. The figure displays the percentages of new (disfluent) and repeated (fluent) statements judged true as a function of likes. Error bars represent standard errors.

In a first model (1a), the level-1 predictor *repetition status* was included. Results demonstrate a significant main effect (*b* = 1.937, *p* < .001), indicating an increased probability of “true” judgments for repeated (vs. new) statements. The odds ratio (*OR*) is 6.94, indicating that the odds for judging “true” (relative to “false”) was 6.94 times larger when a statement was repeated. Thus, we replicated the basic illusory truth effect. In a second model (2a) the predictors *repetition status*, *condition* (control vs. experimental) as well as the interaction between both predictors were included. In addition to the main effect of *repetition status* (*b* = 1.905, *p* < .001; *OR* = 6.72), no significant effects were found (condition: *b* = -0.085, *p* = .431; repetition status x condition: *b* = 0.064, *p* = .796). Table 1 shows all modeling results; see Fig. 1 for the frequencies.

**Table 1:** Multilevel logistic modelling results for the prediction of “true” responses (Model 1a & 2a).

Fixed effects				
	b	SE	z	*p*
Model 1a				
Intercept	0.174	0.054	3.220	0.001**
Repetition Status	1.937	0.124	15.670	< 0.001***
Model 2a				
Intercept	0.216	0.076	2.841	0.005**
Repetition Status	1.905	0.174	10.921	< 0.001***
Condition	− 0.085	0.108	− 0.787	0.431
R.S. x Condition	0.064	0.247	0.259	0.796

*N* = 165. ***p < .001; **p < .01.

An increase in the Akaike information criterion (AIC) and Bayesian information criterion (BIC) indicated a deteriorated model fit for the larger model (AIC = 14080.5; BIC = 14132.9) compared to the more parsimonious model including only repetition status as predictor (AIC = 14077.1; BIC = 14114.5), suggesting that the visibility of likes did not explain substantial variance in truth judgments.

### The illusory truth effect as a function of like count magnitude

For analyses investigating the illusory truth effect as a function of like count magnitude, we used data from the experimental group (*N* = 82), i.e., the participants who received Instagram posts including high (vs. low) like counts. In a first model (1b), the level-1 predictor *repetition status* was included. Results demonstrate a significant main effect (*b* = 1.960, *p* < .001; *OR* = 7.10). In a second model (2b) the predictors *repetition status*, *likes* as well as the interaction between both predictors were included. In addition to the main effect of *repetition status* (*b* = 1.849, *p* < .001; *OR* = 6.35), a significant main effect for *likes* (*b* = -0.304, *p* < .001; *OR* = 0.74) was found, indicating a decreased probability of “true” judgments for posts with low (vs. high) like numbers. As can be seen in Fig. 1, this main effect of likes occurs primarily with new (vs. repeated) statements. However, the interaction effect between repetition status and likes was not significant (*b* = 0.226, *p* = .086). Table 2 shows all results. Figure 1 shows the frequencies underlying these findings.

**Table 2:** Multilevel logistic modelling results for the prediction of “true” responses (Model 1b & 2b).

Fixed Effects				
	b	SE	z	*p*
Model 1b				
Intercept	0.131	0.077	1.708	0.088
Repetition Status	1.960	0.172	11.418	< 0.001***
Model 2b				
Intercept	0.283	0.084	3.383	< 0.001***
Repetition Status	1.849	0.184	10.025	< 0.001***
Likes	− 0.304	0.075	− 4.036	< 0.001***
R.S. x Likes	0.226	0.132	1.715	0.086

*Notes. N* = 82. ***p < .001.

When considered jointly, Akaike information criterion (AIC) and the Bayesian information criterion (BIC) yielded an ambiguous pattern of results, with AIC suggesting a slightly better fit for the model including like counts, whereas BIC favored the more parsimonious model with repetition status as the sole predictor (Model 2b: AIC = 7011.8, BIC = 7079.7; Model 1b: AIC = 7021.5, BIC = 7055.4).

Exploratorily, we also examined whether three brief questions regarding participants’ social media use influenced our results. Specifically, participants indicated (i) whether they use social media platforms such as Instagram or Facebook (agree/disagree), (ii) whether they currently use or have previously used Instagram (agree/disagree), and (iii) how frequently they use social media platforms (e.g., Instagram, Facebook; 1 = not at all to 6 = several times a day). To address this, we included the three social media use variables (questions i and ii effect-coded, question iii grand-mean centered), as well as their interactions with like counts, as additional predictors in Model 2b. The results consistently showed no significant influence of social media use on the effect of like magnitude on truth judgments (all corresponding effects: *p* > .64). In the present sample, social media use was generally high (88% reported using social media platforms; 82% reported current or past Instagram use; usage frequency ranged across the full scale, *M* = 4.39, *SD* = 1.71).

### RT-MPT modeling

To further investigate the role of like magnitude, we conducted an exploratory model comparison of response-time extended multinomial processing tree models (RT-MPTs; Klauer & Kellen^29^) based on data from the experimental group only, as only these participants were exposed to Instagram posts with visible like counts. This class of models extends the classical multinomial processing tree (MPT) framework^30^ by incorporating response times in addition to categorical responses. We used the fit_ertmpt function from the rtmpt package^31,32^; see also^33^ for model estimation and the loo_compare function from the loo package^34^ to compare expected log predictive density (ELPD) of the LOO and WAIC across models. For a goodness-of-fit method *M*: $ELPD = - M_{{IC}} /2$ , were $M_{{IC}}$ is either the LOO-IC or the WAIC.

**Fig. 2:**
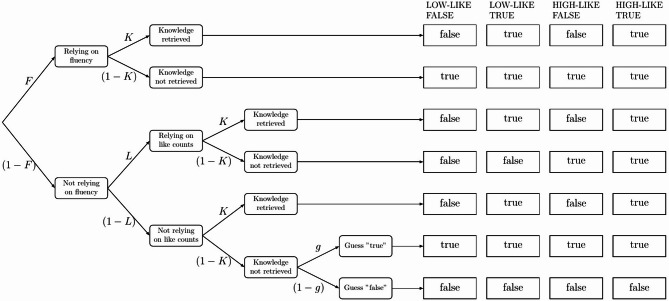
The knowledge-dominant fluency-likes-knowledge model. The figure shows the knowledge-dominant fluency-likes-knowledge MPT model. The letters *F*, *L*, *K*, and *g* denote the probability parameters of the model and stand for the cognitive processes *fluency*, *likes*, *knowledge*, and *guessing*, respectively. The corresponding rounded rectangles represent the latent states that can be reached through these processes. The upper-case bold text in the top-right corner are the conditions in which the model makes different predictions for the responses (“true” and “false”) depicted in the rectangles below these. Only like counts (low vs. high) and factual truth (true vs. false), but not repetition (new vs. repeated), change the predicted responses. However, repetition affects the two parameters *F* and *L*, like counts affects the parameter *L*, and factual truth affects the *K* parameter. That is, there are actually two *F* parameters, four *L* parameters, and two *K* parameters. Knowledge-dominance = if knowledge is successful (i.e., “knowledge retrieved”), it drives the response, no matter what the outcomes of the other processes are. Fluency-dominance over likes = if fluency is successful (i.e., “relying on fluency”) and knowledge not (i.e., “knowledge not retrieved”), it drives the response, irrespective of the like count. In this model, we assume no relying on likes after a fluency experience. The dominance can be read off the expected responses. For example, on the first branch a successful fluency outcome would predict a “true” response and a successful knowledge a correct response. We can see that each response is correct and therefore, knowledge dominates fluency. The order of processes can be read off from the lowest branch in the figure.

MPTs can be understood as probabilistic decision trees. Consider the MPT model shown in Fig. 2 (knowledge-dominant fluency-likes-knowledge). The model includes four cognitive processes that determine how a response is generated. Importantly, despite the term “decision,” these processes are not necessarily conscious. The first process, fluency, determines whether a participant relies on processing fluency when evaluating a statement (with probability F) or not (with probability 1-F). In the present context, fluency was manipulated via repetition. If fluency is used (with probability F), the participant initially tends to respond “true”. However, this tendency may subsequently be overridden by the knowledge process, which determines whether relevant factual knowledge can be retrieved (with probability K) or not (with probability 1-K). If knowledge retrieval is successful, the response is based on factual knowledge (i.e., “true” for objectively true statements and “false” for objectively false statements). If knowledge retrieval fails, the initial fluency-based response tendency remains. If fluency is not used (with probability 1-F), participants may still rely on the number of likes (with probability L) or not (with probability 1-L). We refer to this process as likes. Reliance on likes (L) biases participants toward responding “true” for posts with high numbers of likes (high like condition) and toward responding “false” for posts with low numbers of likes (low like condition). Again, this response tendency may be overridden if factual knowledge is successfully retrieved. If neither fluency nor likes are used and knowledge retrieval fails, the final process, guessing, determines the response. In this case, participants guess “true” (with probability g) or “false” (with probability 1-g). Importantly, the guessing parameter reflects responses given when none of the other processes determine the response and is unrelated to the confidence ratings collected after each truth judgment. However, guessing presumably arises under conditions of uncertainty and may therefore be associated with relatively low confidence ratings, although the guessing parameter itself is conceptually distinct from subjective confidence.

RT-MPTs share the same tree structure as classical MPTs, with each node representing a cognitive process involved in the task. In addition to the process-probability parameters used in MPTs, RT-MPTs additionally incorporate response times. Specifically, RT-MPTs assume that each process outcome (e.g., relying on fluency vs. not relying on fluency) is associated with a time component. In the present analyses, these process completion times (which in principle can be fixed to zero) as well as stimulus encoding and motor response execution times, were freely estimated. Using this model class offers several advantages, including improved estimation of process probabilities, the possibility to test assumptions about process ordering, and the identification of model structures that would otherwise be non-identifiable in standard MPT frameworks^29^.

In our models, we assumed up to four processes: fluency (whether fluency is used as a cue), knowledge (whether factual knowledge is retrieved), likes (whether the number of likes is used as a cue), and guessing, which operates when no other process is engaged. Several theoretical assumptions regarding these processes were evaluated: (i) whether the likes process (i.e., the magnitude of likes) contributes at all (fluency, knowledge, and guessing processes are typically assumed to play a role in the context of the illusory truth effect; see, e.g.,^24,35^, (ii) which process dominates the response when multiple processes are active, and (iii) the order in which processes occur most likely (for example, fluency first, followed by likes, knowledge, and guessing). For the RT-MPT modeling, we excluded trials using Tukey’s fences (k = 3) on the response times. In addition, we excluded trials above 20 s or below 500 milliseconds (if they had not already been removed based on the previous criterion). As a consequence, two subjects were excluded due to an insufficient number of remaining trials (fewer than 64 of 80). The selection of RT-MPT models was based on prior research and practical considerations. Given the large number of possible model variants, we restricted our analyses to the most promising candidates. Based on previous model comparisons (using as yet unpublished data), the fluency-dominant model consistently performed worse than the knowledge-dominant model. Consequently, we included only the most promising variant of the fluency-dominant model. With respect to the reduced versions (no fluency process for new items), prior analyses indicated that they performed substantially worse than all other models. Therefore, we did not evaluate a reduced version of the fluency-dominant model. Decisions regarding the order of processes were also based on previous model comparisons, which suggested that fluency should always be the first process. The ordering of the remaining processes was examined within the present model comparison.

The best-fitting model, according to leave-one-out cross-validation and the widely applicable information criterion (WAIC;^34^, included all four processes. In this model, knowledge dominated fluency, fluency dominated likes, and the order of processes fluency first, followed by likes, then knowledge, and finally guessing (see Fig. 2 for a graphical representation of the model). Across all models, we assumed that repetition influenced the fluency process, like magnitude (and repetition) influenced the likes process, and factual truth influenced knowledge retrieval, such that effects on the corresponding model parameters were admitted. Other processes were assumed not to be affected by experimental manipulations. All model specifications for the tested RT-MPT models (including the relative goodness-of-fit indices) are shown in Table 3. Furthermore, graphical representations of all RT-MPT models can be found in the supplemental material published on OSF. MPT graphics were generated using the R package MPTplot^36^.

**Table 3:** Relative goodness-of-fit for the different RT-MPT model specifications.

Model	Dominance	Order	Red.	#Params	LOO		WAIC	
					$\:\varDelta\:elpd$	SE	$\:\varDelta\:elpd$	SE
1	K > F > L > g	FLKg	No	29	0.0	0.0	0.0	0.0
2	K > F > L > g	FKLg	No	29	− 0.7	1.1	− 0.6	1.0
3	K > L > F > g	FLKg	No	29	− 3.8	2.4	− 4.1	2.3
4	K > L > F > g	FKLg	No	29	− 6.1	2.8	− 6.5	2.7
5	F > K > L > g	FLKg	No	29	− 7.8	5.4	− 7.8	5.2
6	K > F > g	FKg	No	17	− 13.7	6.7	− 13.9	6.6
7	K > F > L > g	FLKg	Yes	26	− 17.0	3.9	− 16.9	3.8
8	K > F > L > g	FKLg	Yes	26	− 17.9	4.0	− 17.2	3.8
9	K > L > F > g	FKLg	Yes	26	− 20.6	4.4	− 20.4	4.3
10	K > L > F > g	FLKg	Yes	26	− 21.3	4.3	− 21.2	4.2
11	K > F > g	FKg	Yes	23	− 54.5	10.7	− 54.7	10.6

*N* = 80. $\:\varDelta\:elpd$ = expected log-predictive density; *SE =* standard error of the $\:\varDelta\:elpd$; K = *knowledge parameter*; F = *fluency parameter*; L = *likes parameter*; g = *guessing parameter*; Dominance = the greater-than symbol indicates dominance – e.g., in model 1, *knowledge* (K) is the most dominant process, followed by *fluency* (F), etc.); Red. = reduced model (“yes” indicates that there is no *fluency* process for new items); #Params = number of group-level parameters; LOO = leave-one-out cross-validation; WAIC = widely-applicable information criterion.

The median probability parameters and their 95% highest density intervals (HDIs; in square brackets) for the best model are: knowledge for true items (K_t_) is 0.006 [0.000003, 0.028], knowledge for false items (K_f_) is 0.003 [0.000006, 0.012], fluency for repeated items (F_r_) is 0.744 [0.638, 0.840], fluency for new items (F_n_) is 0.017 [0.00001, 0.063], likes for repeated high-like items (I_hr_) is 0.033 [0.00004, 0.128], likes for new high-like items (I_hn_) is 0.029 [0.000002, 0.106], likes for repeated low-like items (I_lr_) is 0.091 [0.0007, 0.218], likes for new low-like (I_ln_) is 0.070 [0.0007, 0.145], and guessing (g) is 0.530 [0.472, 0.560]. As expected, given the difficulty of the statements, knowledge parameters were very low. Fluency estimates also exhibited the anticipated pattern, being high for repeated items and very low for new items. The overall influence of likes was small, and the relatively wide HDIs prevent reliable conclusions about differences between the estimates. The guessing was close to 50%, consistent with random responding when no other process was applicable. Table 4 shows all parameter estimates for the best-fitting model.

**Table 4:** Parameter estimates for the best-fitting model.

Parameter	Lower 95% HDI	Median	Upper 95% HDI
F_n_	1.399e − 05	0.017	0.063
F_r_	6.384e − 01	0.744	0.840
g	4.722e − 01	0.530	0.580
K_f_	6.212e − 06	0.003	0.012
K_t_	2.874e − 06	0.006	0.028
L_hn_	1.640e − 06	0.029	0.106
L_hr_	4.246e − 05	0.033	0.128
L_ln_	7.140e − 04	0.070	0.145
L_lr_	6.812e − 04	0.091	0.218

*N* = 80. F_n_ = fluency parameter for new items; F_r_ = fluency parameter for repeated items; g = guessing parameter, K_f_ = knowledge parameter for false items; K_t_ = knowledge parameter for true items; L_hn_ = likes parameter for high-likes new items; L_hr_ = likes parameter for high-likes repeated items; L_ln_ = likes parameter for low-likes new items; L_lr_ = likes parameter for low-likes repeated items; HDI = highest density interval. The lower 95% HDI bound is written in scientific notation, that is 1.5e-05 = 1.5 × 10^− 5^.

A model without the likes process performed substantially worse, indicating that – even though the influence of likes was rather small (between 3% and 9%) – the magnitude of likes play a relevant role in the mechanisms underlying judgments of truth. However, it should also be noted that models with more parameters are inherently more flexible and may be susceptible to overfitting. The model comparison further suggested that fluency is not the dominant process. A model in which likes dominated fluency performed similarly well in terms of ELPD (for LOO and WAIC). In fact, knowledge emerged as the dominant process: whenever knowledge was accessible, it determined the response.

Finally, it should be noted that one model with an alternative ordering of processes performed similarly to the best-fitting model. This model assumed that fluency occurred first, followed by knowledge, then likes, and finally guessing, although it did not outperform the best-fitting model.

### Confidence ratings

Confidence ratings were analyzed using linear mixed models based on maximum likelihood.

### The illusory certainty effect as a function of like visibility

The level-1 predictor *repetition status* was included in a first model (1c). A repetition-based illusory certainty effect was found (*b* = 1.339, *p* < .001), indicating that repetition substantially increased the subjective confidence in truth evaluations. A second model (2c) included the predictors *repetition status*, *condition* as well as the interaction between both predictors. Besides the main effect of *repetition status* (*b* = 1.240, *p* < .001), no significant effects were found (condition: *b* = -0.139, *p* = .279; repetition status x condition: *b* = 0.199, *p* = .317). Table 5 shows all results.

**Table 5:** Multilevel modelling results for the prediction of confidence ratings (Model 1c & 2c).

Fixed effects				
	b	SE	t	*p*
Model 1c				
Intercept	2.965	0.064	46.290	< 0.001***
Repetition Status	1.339	0.099	13.470	< 0.001***
Model 2c				
Intercept	3.034	0.090	33.712	< 0.001***
Repetition Status	1.240	0.140	8.872	< 0.001***
Condition	-0.139	0.128	-1.086	0.279
R.S. x Condition	0.199	0.198	1.004	0.317

*N* = 165. ***p < .001.

An increase in the Akaike information criterion (AIC) and Bayesian information criterion (BIC) indicated a deteriorated model fit for the larger model (AIC = 45055.4; BIC = 45115.3) compared to the more parsimonious model including only repetition status as predictor (AIC = 45053.0; BIC = 45098.0), suggesting that the visibility of likes did not explain substantial variance in confidence ratings.

### The illusory certainty effect as a function of like count magnitude

**Fig. 3:**
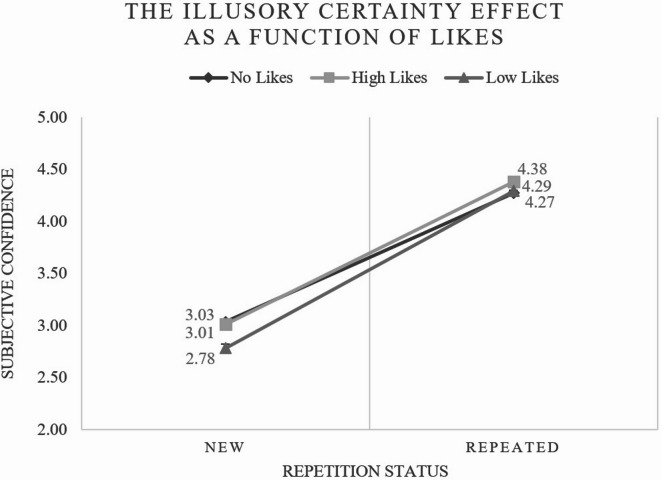
The illusory certainty effect as a function of likes. The figure displays subjective confidence in truth judgments for new (disfluent) and repeated (fluent) statements, as a function of likes. Error bars represent standard errors.

For analyses examining the illusory certainty effect as a function of like count magnitude, we based our analyses on data from the experimental group (*N* = 82); that is, participants who viewed Instagram posts displaying varying numbers of likes. In the first model (1d), *repetition status* was entered as predictor. Again, the results revealed a repetition-induced illusory certainty effect (*b* = 1.439, *p* < .001). In the second model (2d), we included *repetition status*, *like counts*, and their interaction. Besides the main effect of repetition status (*b* = 1.368, *p* < .001), we observed a main effect of like counts (*b* = -0.237, *p* < .001), indicating that, after judging the truth of Instagram posts with low (vs. high) number of likes, participants also showed reduced subjective confidence in their judgments. The interaction between repetition status and like counts was also significant (*b* = 0.143, *p* = .025). Figure 3 provides interpretive context by illustrating the ratings underlying this result. Specifically, it shows that the effect of like counts primarily emerged when participants evaluated new statements – that is, when repetition could not be used as a cue for truth and confidence. Table 6 summarizes all results.

**Table 6:** Multilevel modelling results for the prediction of confidence ratings (Model 1d & 2d).

Fixed effects				
	b	SE	t	*p*
Model 1d				
Intercept	2.896	0.089	32.490	< 0.001***
Repetition Status	1.439	0.134	10.750	< 0.001***
Model 2d				
Intercept	3.014	0.092	32.619	< 0.001***
Repetition Status	1.368	0.138	9.940	< 0.001***
Likes	-0.237	0.048	-4.886	< 0.001***
R.S. x Likes	0.143	0.064	2.237	0.025*

*N* = 82. ***p < .001; *p < .05.

When considered jointly, Akaike information criterion (AIC) and the Bayesian information criterion (BIC) yielded an ambiguous pattern of results, with AIC suggesting a slightly better fit for the model including like counts, whereas BIC favored the more parsimonious model with repetition status as the sole predictor (Model 2d: AIC = 22526.6; BIC = 22601.2; Model 1d: AIC = 22551.8; BIC = 22592.6).

Exploratorily, we also tested whether the three brief indicators of participants’ social media use influenced the results. Participants reported (i) whether they use social media platforms such as Instagram or Facebook (agree/disagree), (ii) whether they currently use or have previously used Instagram (agree/disagree), and (iii) how frequently they use social media platforms (1 = not at all to 6 = several times a day). To examine this, we entered the three social media use variables (questions i and ii effect-coded, question iii grand-mean centered), along with their interactions with like counts, as additional predictors in Model 2d. Across all parameters, social media use showed no significant influence on the effect of like magnitude on truth judgments (all corresponding effects: *p* > .10).

## Discussion

The central aim of the present research was to investigate repetition-based illusions of truth and certainty in more ecologically valid, social-media-like environments, and whether social endorsement cues – specifically the visibility and magnitude of like counts – moderate these effects. The study successfully replicated the illusory truth as well as illusory certainty effect: Repeated statements were more likely to be judged as true and were evaluated with greater confidence than novel statements. The visibility of like counts did not meaningfully alter either of these repetition-driven effects.

However, analyses focusing on the magnitude of like counts revealed a more nuanced pattern. The number of likes appeared to function as a heuristic cue for truth and confidence particularly when participants evaluated novel statements – trials in which repetition as a cognitive shortcut was not available. For confidence ratings, this pattern was reflected not only in a main effect of like count magnitude (in the case of new statements) but also in a substantial interaction between repetition status and like magnitude. That is, the effect of repetition on confidence was even increased in the case of low (vs. high) numbers of likes. For truth judgments the same tendency emerged only descriptively.

These findings suggest that repetition provides a dominant and easily accessible heuristic that can overshadow other judgment-relevant cues. When repetition-based fluency is high, individuals may be less motivated – or less able – to integrate additional indicators such as social endorsement cues into their truth evaluations. In contrast, when statements are presented for the first time and repetition-based signals, such as higher processing fluency and an accompanying feeling of familiarity, are absent, people seem more susceptible to endorsement cues, such as like magnitude, that may inform truth judgments. This interpretation is also consistent with the finding that the mere presence versus absence of like information did not significantly influence truth or confidence judgments, whereas the magnitude of visible likes did. If social endorsement cues constituted a more dominant cue than repetition, one would also expect participants to rely primarily on likes rather than repetition whenever like information was available. This should have resulted in a larger repetition-based truth effect in the control group (posts without likes) compared to the experimental group (posts with visible likes). Taken together, however, the present findings point in the opposite direction, suggesting that repetition constitutes a more dominant and readily accessible cue than social endorsement information. Future research may investigate more directly how individuals attend to and use social endorsement cues. For example, eye-tracking approaches may help determine whether participants selectively attend to like information depending on the availability of other heuristic cues such as information repetition.

To further examine the underlying cognitive mechanisms, we additionally fitted several response-time multinomial processing tree models (RT-MPTs). These models break down the mechanism underlying truth judgments into distinct cognitive processes – namely, influences of processing fluency, knowledge, likes, and guessing – and allow us to test how repetition and like counts contribute to subjective truth. Model comparisons revealed that the best-fitting model included all four processes. Although the likes parameters were numerically small and estimated with considerable uncertainty, removing the likes parameters led to a substantially worse model fit, suggesting that the magnitude of likes, although weak, was processed and contributed to truth evaluations. Importantly, the best-fitting model further suggested that fluency-based processes dominated like-based processes. At the same time, the modeling suggested that whenever knowledge was accessible, it tended to dominate both fluency- and like-based pathways to truth judgments, consistent with the idea that knowledge retrieval, when available, can override heuristic cues such as fluency and likes (see also^24,37^). However, consistent with the intentionally difficult statement material used in the present study, the estimated knowledge parameters were overall very small.

Taken together, the pattern of results is well in line with recent demonstrations that social endorsement cues can influence truth evaluations in digital environments^18^. Beyond this, our findings extend previous work by showing that like counts may not only affect truth judgments but also subjective confidence in truth evaluations. This is noteworthy because confidence is not merely a secondary metric but has been shown to predict downstream behaviors such as further information seeking. Thus, even subtle influences on confidence may have practical implications for how individuals engage with (mis-)information in social media environments. At the same time, the present findings shed light on the boundary conditions of such effects of endorsement cues. The strong and consistent repetition effects observed within our study suggest that information repetition offers a particularly powerful pathway to perceived truth and subjective certainty. Thus, while social endorsement cues can shape truth judgments, their influence appears limited when a more potent internal cue, such as repetition, is available.

In addition, we explored whether participants’ social media use shaped the influence of like magnitude on subjective truth and confidence judgments. Across both outcome variables, we found no systematic effects of social media use. However, given the generally high level of social media engagement within our sample, these null findings should be interpreted with some caution, as limited variability in usage may have restricted the potential to detect such influences.

The present study has several further limitations. First, although our design aimed to approximate social media environments and thereby increase ecological validity, the posts still differed from those encountered in everyday use. We intentionally removed visual content and grayed out backgrounds to avoid confounding influences, which helped isolate textual (i.e., repetition of written information) and endorsement cues but reduced the richness of our stimuli relative to real social media posts. Second, although our Prolific sample spanned a broad age range and varied in gender, it still reflects a specific cultural and demographic context. As such, generalizability to other populations with different cultural backgrounds or media habits remains uncertain. Third, we intentionally used highly difficult statement material in order to investigate the influence of repetition and social endorsement cues under conditions of uncertainty. Accordingly, factual knowledge was likely to play only a very limited role in participants’ judgments, which is also reflected in the very low knowledge parameter estimates observed in the exploratory RT-MPT analyses. Although this design was well suited to examine the use of heuristic cues such as repetition and likes, it limits conclusions regarding the role of factual knowledge in repetition- and social endorsement-based effects. Future research should therefore systematically investigate how factual knowledge interacts with repetition and social endorsement cues using materials that vary in objective difficulty and knowledge accessibility. Finally, our design focused on single-exposure truth evaluations. Future studies could employ repeated- or longitudinal-exposure paradigms to examine how repetition and social endorsement cues interact over time, particularly in environments where information recirculates frequently. In this context, future work could also vary not only the number of repetitions and number of truth-judgment prompts but also the retention interval between exposures, as these factors may influence how fluency and endorsement cues interact over time.

## Data Availability

The data, code, and graphical representations of all RT-MPT models are publicly accessible at https://osf.io/9x2wv/overview?view_only=3c29dd62348944ec9e153e1ee331a22a.

## Appendix A: Exploratory analyses across all levels of social endorsement information (no likes, low likes, high likes)

Following a reviewer suggestion, we additionally estimated exploratory mixed-effects models across the full sample including all three levels of social endorsement information (no likes, low likes, high likes).

For truth judgments, the analyses revealed a significant main effect of repetition (*b* = 1.895, *p* < .001), indicating an increased probability of “true” judgments for repeated statements. No significant difference emerged between the control group (without visible likes) and the high-like condition from the experimental group (*b* = 0.067, *p* = .550). In contrast, posts with low like counts in the experimental group were associated with reduced truth judgments relative to the control group (*b* = -0.237, *p* = .042). Neither the interaction between repetition and the high-like condition (*b* = -0.024, *p* = .925) nor the interaction between repetition and the low-like condition (*b* = 0.201, *p* = .431) reached significance.

For confidence ratings, the same overall pattern emerged. Repetition significantly increased subjective confidence (*b* = 1.240, *p* < .001). Again, no significant difference emerged between the control group (without visible likes) and the high-like condition from the experimental group (*b* = -0.020, *p* = .876). In contrast, posts with low like counts in the experimental group were associated with reduced confidence relative to the control group (*b* = -0.257, *p* = .049). Neither the interaction between repetition and the high-like condition (*b* = 0.128, *p* = .526) nor the interaction between repetition and the low-like condition (*b* = 0.270, *p* = .180) was significant.

## References

[1] Brashier, N. M. & Marsh, E. J. Judging truth. *Ann. Rev. Psychol.* **71**, 499–515. 10.1146/annurev-psych-010419-050807 (2020). 31514579 10.1146/annurev-psych-010419-050807

[2] Reber, R. & Unkelbach, C. The epistemic status of processing fluency as source for judgments of truth. *Rev. Philos. Psychol.* **1**, 563–581. 10.1007/s13164-010-0039-7 (2010). 22558063 10.1007/s13164-010-0039-7 PMC3339024

[3] Dechêne, A., Stahl, C., Hansen, J. & Wänke, M. The Truth About the Truth: A Meta-Analytic Review of the Truth Effect. *Personality Social Psychol. Rev.* **14** (2), 238–257. 10.1177/1088868309352251 (2010). 10.1177/1088868309352251 20023210

[4] Hasher, L., Goldstein, D. & Toppino, T. Frequency and the conference of referential validity. *J. Verbal Learn. Verbal Behav.* **16** (1), 107–112. 10.1016/S0022-5371(77)80012-1 (1977).

[5] Reber, R. & Schwarz, N. Effects of perceptual fluency on judgments of truth. *Conscious. Cogn.* **8** (3), 338–342. 10.1006/ccog.1999.0386 (1999). 10487787 10.1006/ccog.1999.0386

[6] Arkes, H. R., Boehm, L. E. & Xu, G. Determinants of judged validity. *J. Exp. Soc. Psychol.* **27** (6), 576–605. 10.1016/0022-1031(91)90026-3 (1991).

[7] Stump, A., Schumacher, L., Voss, A. & Klauer, K. C. Cognitive processes underlying the repetition-based truth effect: A diffusion model study. *Journal of Experimental Psychology: Learning, Memory, and Cognition.* Advance online publication. 10.1037/xlm0001628 (2026). 10.1037/xlm0001628 42113201

[8] Topolinski, S., Likowski, K. U., Weyers, P. & Strack, F. The face of fluency: Semantic coherence automatically elicits a specific pattern of facial muscle reactions. *Cognition Emot.* **23** (2), 260–271. 10.1080/02699930801994112 (2009).

[9] Winkielman, P. & Cacioppo, J. T. Mind at ease puts a smile on the face: Psycho-physiological evidence that processing facilitation elicits positive affect. *J. Personal. Soc. Psychol.* **81** (6), 989–1000. 10.1037/0022-3514.81.6.989 (2001). 11761320

[10] Winkielman, P., Schwarz, N., Fazendeiro, T. & Reber, R. The hedonic marking of processing fluency: Implications for evaluative judgment. In (eds Musch, J. & Klauer, K. C.) The psychology of evaluation: Affective processes in cognition and emotion (189–217). Lawrence Erlbaum Associates. (2003).

[11] Stump, A., Rummel, J. & Voss, A. Is it all about the feeling? Affective and (meta-) cognitive mechanisms underlying the truth effect. *Psychol. Res.* **86**, 12–36. 10.1007/s00426-020-01459-1 (2022). 33484352 10.1007/s00426-020-01459-1 PMC8821071

[12] Stump, A., Wüstenberg, T., Rouder, J. N. & Voss, A. The face of illusory truth: Repetition of information elicits affective facial reactions predicting judgments of truth. *Cogn. Affect. Behav. Neurosci.* **25**, 769–782. 10.3758/s13415-025-01266-4 (2025). 40011403 10.3758/s13415-025-01266-4 PMC12130120

[13] Garcia-Marques, T., Mackie, D. M., Claypool, H. M. & Garcia-Marques, L. Positivity can cue familiarity. *Pers. Soc. Psychol. Bull.* **30** (5), 585–593. 10.1177/0146167203262856 (2004). 15107158 10.1177/0146167203262856

[14] Klauer, K. C. & Musch, J. Affective priming: Findings and theories. In (eds Musch, J. & Klauer, K. C.) The psychology of evaluation: Affective processes in cognition and emotion (7–49). Lawrence Erlbaum Associates (2003).

[15] Wentura, D. Dissociative affective and associative priming effects in the lexical decision task: Yes versus no responses to word targets reveal evaluative judgment tendencies. *J. Experimental Psychology: Learn. Memory Cognition*. **26** (2), 456–469. 10.1037/0278-7393.26.2.456 (2000). 10.1037//0278-7393.26.2.456 10764106

[16] Stump, A., Wüstenberg, T. & Voss, A. Stop frowning, it´s true: reduced corrugator activity indicates increased positive affect after judging information as true. *Cogn. Emot.* 1–10. 10.1080/02699931.2026.2614304 (2026). 10.1080/02699931.2026.2614304 41533842

[17] Stump, A., Voss, A. & Rummel, J. The illusory certainty: Information repetition and impressions of truth enhance subjective confidence in validity judgments independently of the factual truth. *Psychol. Res.* **88**, 1288–1297. 10.1007/s00426-024-01956-7 (2024). 38526581 10.1007/s00426-024-01956-7 PMC11143013

[18] Luo, M., Hancock, J. T. & Markowitz, D. M. Credibility Perceptions and Detection Accuracy of Fake News Headlines on Social Media: Effects of Truth-Bias and Endorsement Cues. *Communication Res.* **49** (2), 171–195. 10.1177/0093650220921321 (2022).

[19] Jalbert, M., Schwarz, N. & Newman, E. Only half of what i’ll tell you is true: Expecting to encounter falsehoods reduces illusory truth. *J. Appl. Res. Memory Cognition*. **9** (4), 602–613. 10.1016/j.jarmac.2020.08.010 (2020).

[20] Maas, C. J. M. & Hox, J. J. Sufficient sample sizes for multilevel modeling. *Methodology: Eur. J. Res. Methods Behav. Social Sci.* **1** (3), 86–92. 10.1027/1614-2241.1.3.86 (2005).

[21] Paccagnella, O. Sample size and accuracy of estimates in multilevel models: New simulation results. *Methodology: Eur. J. Res. Methods Behav. Social Sci.* **7** (3), 111–120. 10.1027/1614-2241/a000029 (2011).

[22] Moineddin, R., Matheson, F. I. & Glazier, R. H. A simulation study of sample size for multilevel logistic regression models. *BMC Med. Res. Methodol.* **7**, 34. 10.1186/1471-2288-7-34 (2007). 17634107 10.1186/1471-2288-7-34 PMC1955447

[23] Unkelbach, C. & Stahl, C. A multinomial modeling approach to dissociate different components of the truth effect. *Conscious. Cogn.* **18** (1), 22–38. 10.1016/j.concog.2008.09.006 (2009). 18980847 10.1016/j.concog.2008.09.006

[24] Shechter, A. & Klauer, K. C. Rethinking Knowledge’s Impact on the Illusory Truth Effect. *Personality and Social Psychology Bulletin*. Advance online publication. 10.1177/01461672251403392 (2026). 10.1177/01461672251403392 41460245

[25] Nadarevic, L. *Die Wahrheitsillusion [The illusory truth effect]* (Verlag Dr. Köster, 2010).

[26] Henninger, F., Shevchenko, Y., Mertens, U. K., Kieslich, P. J. & Hilbig, B. E. lab.js: A free, open, online study builder. *Behav. Res. Methods*. **54** (2), 556–573. 10.3758/s13428-019-01283-5 (2022). 34322854 10.3758/s13428-019-01283-5 PMC9046347

[27] Bates, D., Mächler, M., Bolker, B. & Walker, S. Fitting linear mixed-effects models using lme4. *J. Stat. Softw.* **67** (1), 1–48. 10.18637/jss.v067.i01 (2015).

[28] Kuznetsova, A., Brockhoff, P. B. & Christensen, R. H. B. lmerTest Package: Tests in Linear Mixed Effects Models. *J. Stat. Softw.* **82** (13), 1–26. 10.18637/jss.v082.i13 (2017).

[29] Klauer, K. C., & Kellen, D. Rt-mpts: process models for response-time distributions based on multinomial processing trees with applications to recognition memory. *Journal of Mathematical Psychology*, **82**, 111–130. 10.1016/j.jmp.2017.12.003 (2018).

[30] Riefer, D. M. & Batchelder, W. H. Multinomial modeling and the measurement of cognitive processes. *Psychol. Rev.* **95** (3), 318 (1988).

[31] Hartmann, R., Johannsen, L. & Klauer, K. C. rtmpt: An R package for fitting response-time extended multinomial processing tree models. *Behav. Res. Methods*. **52** (3), 1313–1338. 10.3758/s13428-019-01318-x (2020). 32377974 10.3758/s13428-019-01318-x

[32] Hartmann, R. & Klauer, K. C. Extending RT-MPTs to enable equal process times. *J. Math. Psychol.* **96**, 102340. 10.1016/j.jmp.2020.102340 (2020).

[33] Klauer, K. C., Hartmann, R. & Meyer-Grant, C. G. RT-MPTs: Process models for response-time distributions with diffusion-model kernels. *J. Math. Psychol.* 120–121. 10.1016/j.jmp.2024.102857 (2024).

[34] Vehtari, A., Gelman, A. & Gabry, J. Practical Bayesian model evaluation using leave-one-out cross-validation and WAIC. *Stat. Comput.* **27** (5), 1413–1432. 10.1007/s11222-016-9696-4 (2016).

[35] Fazio, L. K., Brashier, N. M., Payne, B. K. & Marsh, E. J. Knowledge does not protect against illusory truth. *Journal Experimental Psychology: General*. **144** (5), 993–1002. 10.1037/xge0000098 (2015). 10.1037/xge0000098 26301795

[36] Hartmann, R. *MPTplot: Draw multinomial processing tree plots with TikZ (R package version 0.0.3)*. GitHub. (2025). https://github.com/RaphaelHartmann/MPTplot

[37] Meyer-Grant, C. G., Shechter, A., & Klauer, K. C. The disputed role of knowledge in truth illusions: A critical test based on comparative judgments. *Cognition*, **274**, 106572. 10.1016/j.cognition.2026.106572 (2026). 10.1016/j.cognition.2026.106572 42166847

